# 2019 novel coronavirus of pneumonia in Wuhan, China: emerging attack and management strategies

**DOI:** 10.1186/s40169-020-00271-z

**Published:** 2020-02-20

**Authors:** Jun She, Jinjun Jiang, Ling Ye, Lijuan Hu, Chunxue Bai, Yuanlin Song

**Affiliations:** Department of Pulmonary Medicine, Zhongshan Hospital, Fudan University, 180 Feng Lin Road, Shanghai, 200032 China

**Keywords:** 2019-nCoV, Transmission, Isolation, Respiratory and eye protection, Hand hygiene

## Abstract

An ongoing outbreak of 2019-nCoV pneumonia was first identified in Wuhan, Hubei province, China at the end of 2019. With the spread of the new coronavirus accelerating, person-to-person transmission in family homes or hospitals, and intercity spread of 2019-nCoV occurred. At least 40,261 cases confirmed, 23,589 cases suspected, 909 cases death and 3444 cases cured in China and worldwide 24 countries confirmed 383 cases being diagnosed, 1 case death in February 10th, 2020. At present, the mortality of 2019-nCoV in China is 2.3%, compared with 9.6% of SARS and 34.4% of MERS reported by WHO. It seems the new virus is not as fatal as many people thought. Chinese authorities improved surveillance network, made the laboratory be able to recognize the outbreak within a few weeks and announced the virus genome that provide efficient epidemiological control. More comprehensive information is required to understand 2019-nCoV feature, the epidemiology of origin and spreading, and the clinical phenomina. According to the current status, blocking transmission, isolation, protection, and alternative medication are the urgent management strategies against 2019-nCoV.

## Background

**Fig. 1:**
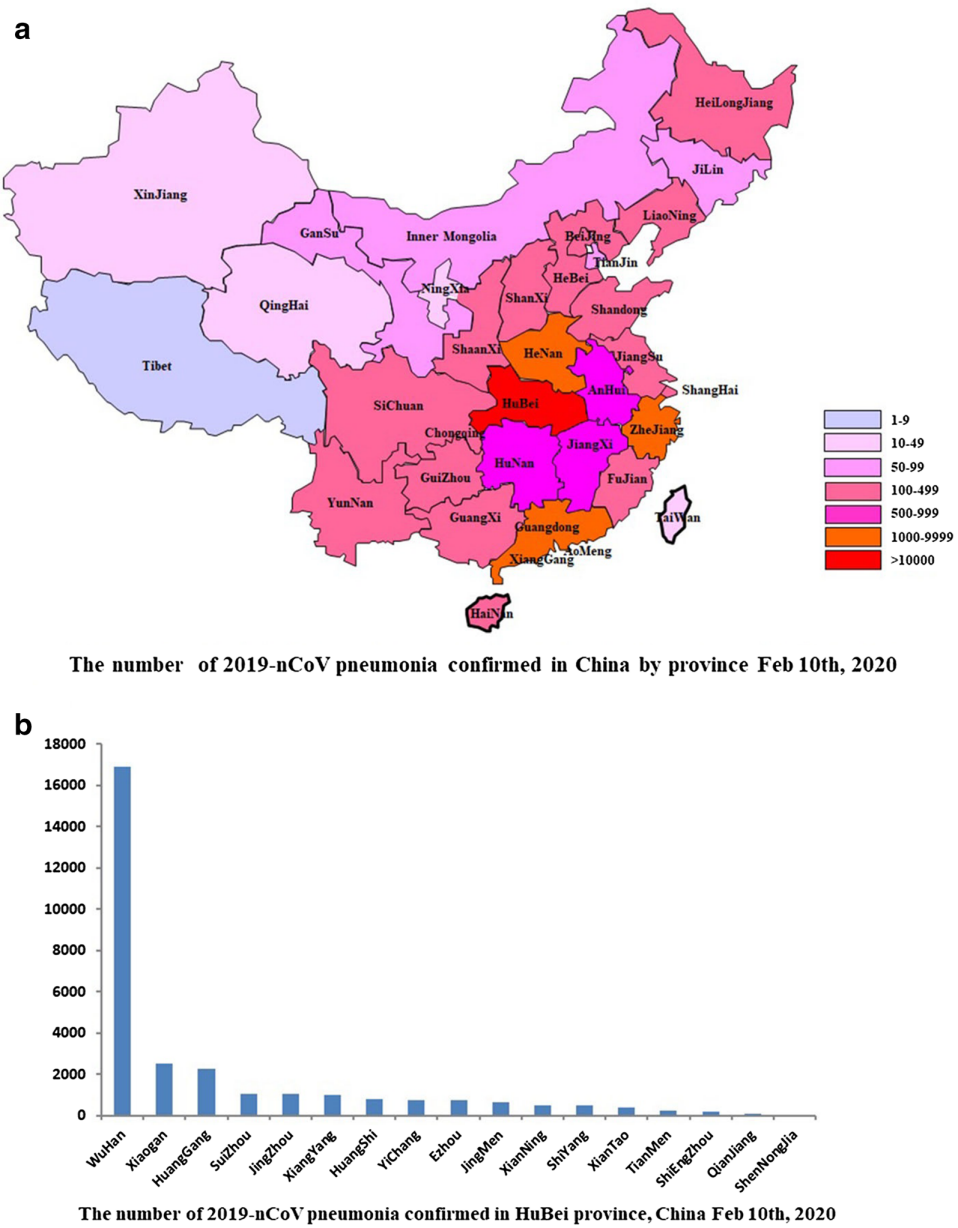
**a** The number cases of 2019-nCoV pneumonia confirmed in China by province Feb 10th, 2020; **b** The number cases of 2019-nCoV pneumonia confirmed in Hubei province, China Feb 10th, 2020

**Fig. 2:**
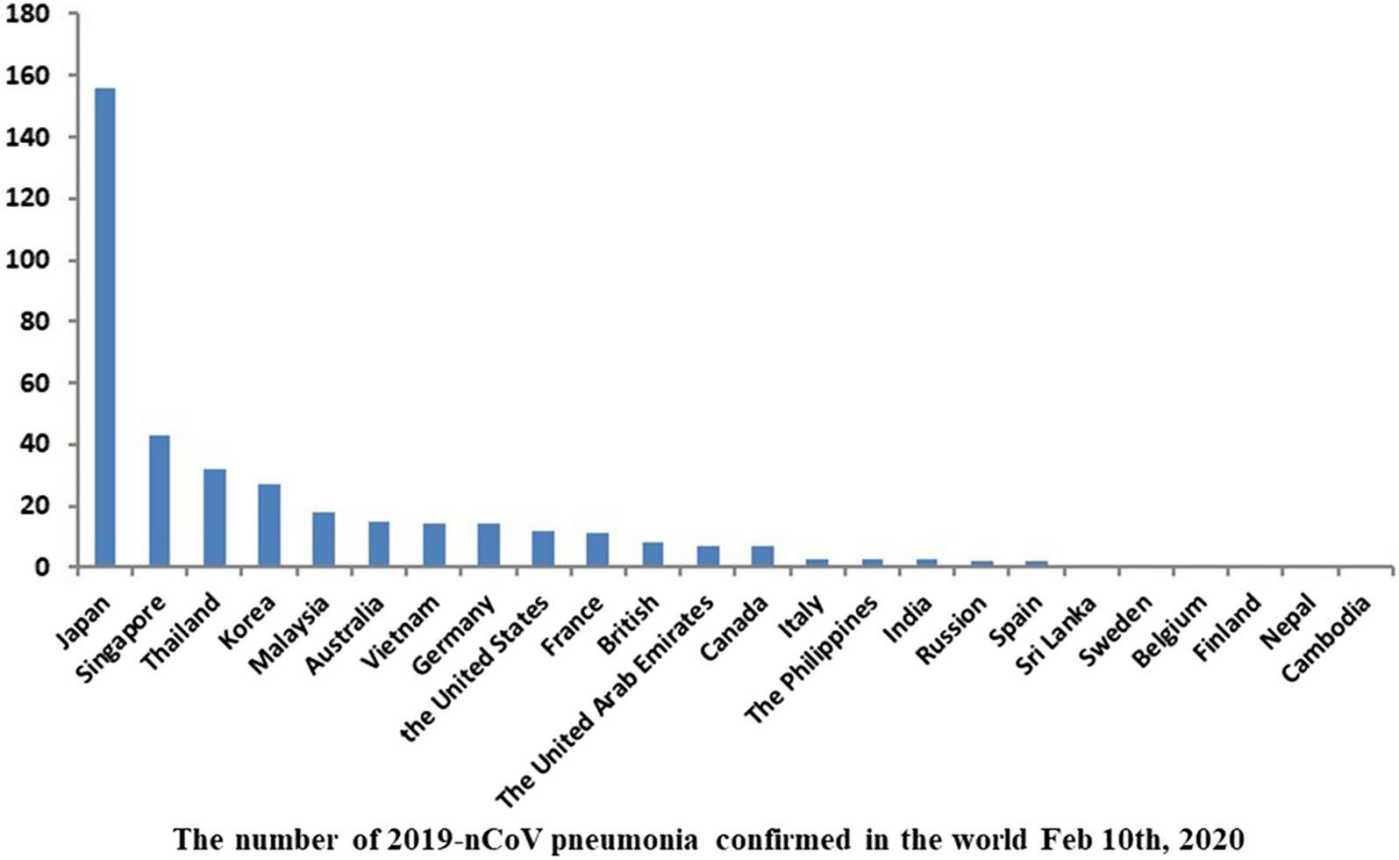
The number of 2019-nCoV pneumonia confirmed in the world 10 Feb 2020

An outbreak of novel coronavirus pneumonia is ongoing, called 2019-nCoV, was first identified in Wuhan, Hubei province, China at the end of 2019 [1, 2]. As of February 10th, 2020, at least 40,261 cases confirmed, 23,589 cases suspected, 909 cases death and 3444 cases were cured in China (Fig. 1) [3]. 24 countries (Fig. 2) such as Japan, Singapore, Thailand, Korea, and the United States have 383 cases being diagnosed, with 1 case death so far [3]. Although Chinese authorities improved surveillance network, made the laboratory be able to recognize the outbreak within a few weeks and announced the virus genome that provide efficient epidemiological control, World Health Organization (WHO) assessed the risk as ‘very high’ in China and ‘high’ in global level in the coming weeks [4], and declared the public health emergency of international concern (PHEIC) over the global outbreak of 2019-nCoV in January 31, 2020.

Based on the current epidemiological survey and data, more comprehensive information is required to understand 2019-nCoV feature, epidemiology of the outbreak including the source, transmission, extent of infection, and the clinical picture. Further strategies are required to determine according to the current status.

## Epidemiology

### 2019-nCoV features

Coronaviruses are enveloped, positive-sense, single stranded RNA viruses that are distributed broadly among humans, other mammals, and birds, which cause respiratory, enteric, hepatic, and neurologic diseases [5]. Six coronavirus species are known to cause human disease. Four viruses including hCoV-229E, OC43, NL63, and HKU1 are prevalent and typically cause mild respiratory diseases [6]. The two novel fatal coronaviruses emerge periodically in different areas, severe acute respiratory syndrome coronavirus (SARS-CoV) in 2002 and Middle East respiratory syndrome coronavirus (MERS-CoV) in 2012. Given the high prevalence and wide distribution of coronaviruses, the genetic diversity and frequent recombination of genomes, and increasing human and animal activities, novel coronaviruses are likely to emerge periodically in humans owing to frequent cross-species infections and occasional spillover events [7, 8].

As Zhu et al. reported that the identified 2019-nCoV genome has been sequenced are phylogenetically the closest to certain beta-coronaviruses detected in bats, belonging to the sarbecovirus subgenus of coronaviridae family [1], and these results in conjunction with other reports show that it is 75–80% identical to the SARS-CoV [9, 10] and 40% identical to the MERS-CoV. It can be propagated in the same cells for growing SARS-CoV and MERS-CoV. Notably, 2019-nCoV grows better in primary human airway epithelial cells than in standard tissue-culture cells, unlike SARS-CoV or MERS-CoV. It appears that 2019-nCoV uses the same cellular receptor hACE2 (human angiotensin-converting enzyme 2) as SARS-CoV [11], it seems the transmission may develop after signs of lower respiratory tract disease.

### Origin

The origin, spread and virulence of 2019-nCoV remain largely unknown. Most of early infected patients were linked to Huanan seafood wholesale market in Wuhan, China. However,there were 13 of the 41 cases had no link to the marketplace [2]. Most importantly, in the earliest case, the patient became ill on the 1st December 2019 and had no reported access to the seafood market. And no epidemiological link was found between the first patient and later cases. It seems that the seafood market is not the only origin of the virus. The virus probably came into the market place first then it went out of there [12]. Analyses of blood samples in China from people and animals from other animal markets may reveal a clear picture of where the 2019-nCoV originated.

### Spreading

An increasing number of cases evidenced the 2019-nCoV have the ability to transmit among humans [13, 14]. To date, no research found the special susceptible population of the new virus seems like SARS [15], 2019-nCoV is easily transmissible in human generally, but disease severity is not correlated to transmission efficiency [16]. According to the Chinese Center for Disease Control and Prevention (China CDC) reported that laboratory tests ruled out SARS-CoV, MERS-CoV, influenza, avian influenza, adenovirus and other common respiratory pathogens. CDC considered the 2019-nCoV as a possible pathogen causing the outbreak [16]. The 2019-nCoV can cause severe illness in old patients with comorbidities and transmit readily among people [17]. At present, the mortality of 2019-nCoV in China is 2.3% [3], compared with 9.6% of SARS and 34.4% of MERS reported by WHO [16, 18]. Based on the current data, the new virus is not as fatally as many people thought.

The climate of temperature, relative humidity, and wind velocity should also be attention to the transmission. 2019-nCoV pneumonia emerging attacks in the cold seasonal nature akin to viruses such as SARS and influenza [19].

## Clinical features

Based on the current epidemiological survey, most individuals had a history of close contact to a patient who had 2019-nCoV infection or a history of travel from Wuhan City or Hubei province, China. The incubation period is generally 3–7 days (within 14 days) [20].

### Symptoms

The symptoms of 2019-nCoV infection were nonspecific. The most common symptoms were onset of fever, generalized weakness and dry cough. Some patients had headache and/or myalgia, but upper respiratory symptoms such as runny nose were rare [20]. Diarrhea was often identified, which had been reported 10.6% in SARS and up to 30% in MERS [21]. More than half of patients developed shortness of breath, the median duration from disease onset to dyspnea was 8 days [2]. Patients infected with 2019-nCoV might develop acute respiratory distress syndrome (ARDS), followed by septic shock, refractory metabolic acidosis and coagulation dysfunction, if the disease could not be controlled [20].

Notably, some patients were afebrile or confirmed biologically to have an asymptomatic infection [21]. These cryptic cases of walking pneumonia might serve as a possible source to propagate the outbreak. Further studies on the epidemiological significance of these asymptomatic cases are warranted.

### Laboratory findings

The blood cell counts of patients showed total white blood cells, lymphocyte, and platelet were lower than the average with extended activated thromboplastin time, increased C-reactive protein and muscle enzyme level. D-dimer level were higher and lymphocyte decrease progressively, if the disease had aggravation. The cytokine storm such as IL1B, IL1RA, IL7, IL8 could be associated with disease severity [2, 21].

**Fig. 3:**
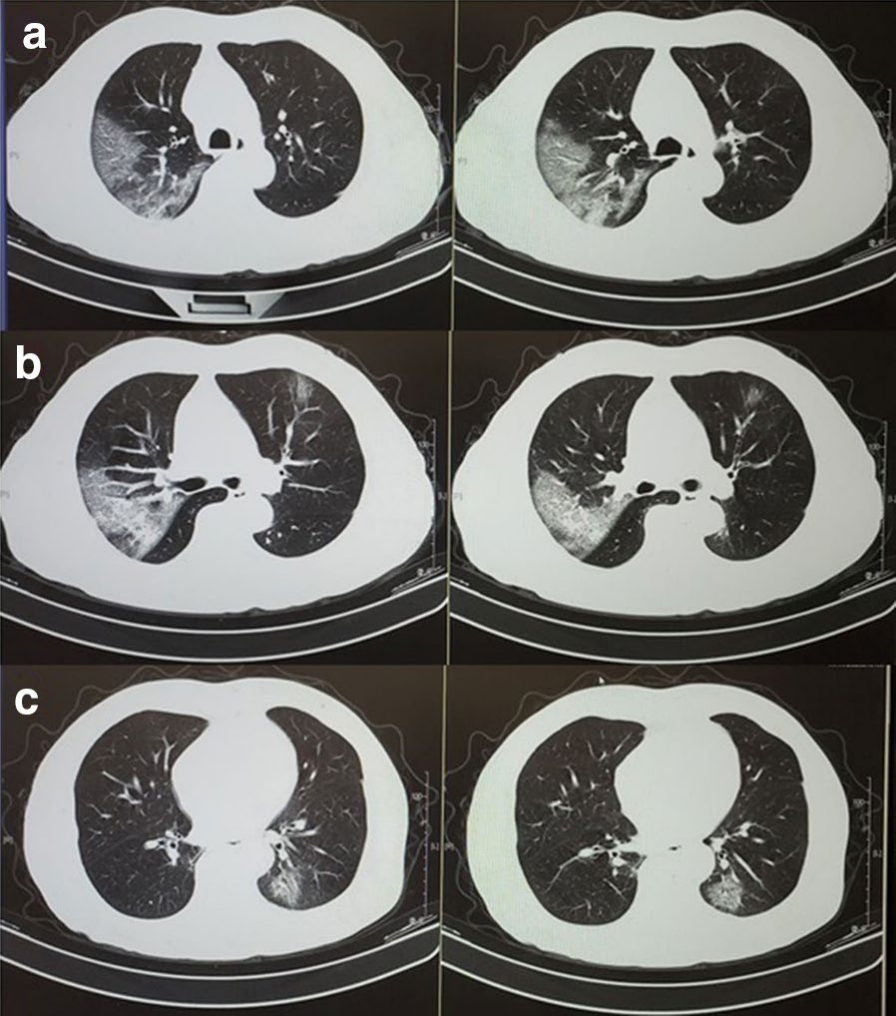
The multifocal ground glass changes on chest CT scan were typical of viral pneumonia in one infected 2019-nCoV patients. **a** shadow in right upper lobe; **b** shadow in bilateral lobe; **c** shadow in left lower lobe

The multifocal ground glass changes on chest CT scan were typical of viral pneumonia [Fig. 3]. If the disease continued to develop, the bilateral multiple lobular and subsegmental areas of consolidation would be found on chest CT scan [2, 21]. The lungs of aged patients showed more diffuse and extensive imaging than those of the younger patients [20].

### Microbiological testing

Nasopharyngeal swab or sputum samples of patients were available for testing by specific RT-PCR assays for 2019-nCoV to detect the highly conserved RdRp and variable S gene. The cycle threshold values of the sputum samples were 8–13 cycles earlier than those of throat swabs, indicating higher viral loads detected in the lower respiratory tract. It is consistent with the observations in patients with MERS who had higher viral loads in lower respiratory tract samples than in upper respiratory tract samples [22].

Serum samples for 2019-nCoV which might indicate some virus spillover from the more severely infected lung into the systemic circulation, as previously reported in patients with SARS [23]. However, the first case of 2019-nCoV in the United States report the stool and respiratory specimens from the patients tested positive by RT-PCR for 2019-nCoV, whereas the serum remained negative [24].

Importantly, the ground glass changes on chest CT scan appeared earlier than the positive for RT-PCR test in some cases. Repeat testing of nasopharyngeal swab or sputum samples are recommended in clinical suspected cases with an initially negative result.

## Management strategy

### Blocking transmission

Learning from the SARS outbreak, which started as animal-to-human transmission during the first phase of the epidemic, all game meat trades should be terminate to prevent this portal of transmission. At the same time, we could not ignore the environmental assessments at the seafood market and investigations to identify the pathogen causing the outbreak.

Person-to-person transmission was efficient and super-spreading events had led to major outbreaks in public gathering places. The severity of disease is an important indirect factor helps to identify those who had been infected. If infection does not cause serious disease or asymptomatic infection, infected people probably end up in health care centers. Instead, they would go to work and travel, thereby potentially spreading the virus to their contacts [16]. Recent epidemiological survey and studies showed the patients who did not travel to Wuhan became infected with the virus after several days of contact with the family members. None of the family members had contacts with Wuhan markets or animals, neither had visited a Wuhan hospital [21]. Person-to-person transmission in family homes or hospital, and intercity spread of 2019-nCoV are occurring, and therefore vigilant control measures are warranted at the whole stage of the epidemic.

### Isolation

Fortunately, with the spread of the new coronavirus accelerating, Chinese authorities responded not only treating the patients and isolating new cases as identified, tracing the contact, but also extended travel restrictions to 48 million people in hardest-hit Hubei province, banned inter-province buses to Beijing and canceled tour group travel abroad [25, 26]. However it is not completely under control, people with symptoms of pneumonia reported travel history to Wuhan have been identified at international airports over the past week.

It is crucial to isolate patients, trace and quarantine contacts as early as possible because asymptomatic infection began to appear [21]. Most importantly, the extent of interhuman transmission needs to be determined. Transmission of SARS-CoV and MERS-CoV occurred to a large extent by means of superspreading events [27, 28]. Superspreading events have been implicated in 2019-nCoV transmission, so educate the public on both food and personal hygiene, and compliance to infection isolation to prevent super-spreading events deserves highly attention.

### Protection

Transmission of 2019-nCoV probably occurs through spreading airborne and contact. Aerosol and fecal–oral transmission remain unclear [24]. Public health measures, including quarantining in the community as well as timely diagnosis and strict adherence to universal precautions in health care settings [29], were critical in reducing the transmission of 2019-nCoV.

For healthcare personnel, to minimize the chance of exposures to 2019-nCoV needs to follow the standard of contact and airborne precautions, personal protection including gloves, gowns, respiratory protection, eye protection, and hand hygiene. Some procedures performed on 2019-nCoV infected patients could generate infectious aerosols, e.g., nasopharyngeal specimen collection, sputum induction, and open suctioning of airways should be performed cautiously. If performed, these procedures should take place in an airborne infection isolation room, and personnel should use respiratory and eye protection, and hand hygiene [30]. In addition, management of environmental infection control including laundry, food service utensils, and medical waste should also be performed. Artificial Intelligence (AI), alternative selection to reducing infection for medical personnel, should be explored (Joint developed by Respiratory Research Institution of Zhongshan Hospital, Fudan University and RealMax Ltd Co), which will be benefit for remote guidance of practices.

### Alternative medication

No antiviral treatment for coronavirus infection has been proven to be effective. Previous studies showed the combination of lopinavir and ritonavir may be beneficial for SARS-CoV and MERS-CoV infected patients [31, 32]. Treatment with intravenous remdesivir (a novel nucleotide analogue prodrug in development) showed significant improvement for the first case in US [24]. A trial has been initiated quickly to assess the efficacy and safety of remdesivir in patients hospitalized with 2019-nCoV infection. Recently, a potent binding of 2019-nCoV spike protein by a SARS-CoV specific human monoclonal antibody were under investigation [33].

As the cytokine storm was observed in severe 2019-nCoV infection patients, low dose corticosteroids has been used to treat the patients for possible benefit by reducing inflammatory-induced lung injury. However, corticosteroids did not reduce the mortality for SARS-CoV and MERS-CoV infection by WHO interim guidance [34, 35].

Treatment regiments were classified into three categories depends on the severity of the disease: (1) For mild to moderate disease, the major treatment is supportive therapy [21]; (2) for severe disease, oxygen inhalation through mask, high nasal oxygen flow inhalation, or non-invasive ventilation is needed. Careful and dynamic evaluation of patients oximeter and Chest imaging as well as laboratory examination is important; (3) for very severe disease, protective mechanical ventilation after tracheal intubation is required, and prone position ventilation followed if P/F ratio not improved, and eventually extracorporeal membrane oxygenation (ECMO) might be implemented if prone position plus mechanical ventilation did not work. Notably, the anxiety and depression of patients need to be consideration. We should not only pay attention to disease treatment, but also the mental issues of patients.

In addition, some traditional Chinese medicine (TCM), such as Snow Lotus (Saussuea involucrata), LianHuaQingWen [36], LiuShenWan [37] might be beneficial for coronavirus infection treatment through immunity enhancement. Further evidence is needed to assess the effect of TCM treatment for patients infected with 2019-nCoV.

## Conclusions

2019-nCoV pneumonia are emerging attack at China and worldwide in the winter of 2019-2020. The identified 2019-nCoV genome has been sequenced the closest to some beta-coronaviruses detected in bats. Person-to-person transmission in family homes or hospital, and intercity spread of 2019-nCoV are occurring. At present, the mortality of 2019-nCoV in China is 2.3%, compared with 9.6% of SARS and 34.4% of MERS reported by WHO. It seems the new virus is not as fatally as many people thought. The most common symptoms were onset of fever, generalized weakness and dry cough. Notably, some patients were afebrile or confirmed biologically to have an asymptomatic infection. And the ground glass changes on chest CT scan were earlier than the positive for RT-PCR test in some cases. Repeat testing of nasopharyngeal swab or sputum samples are recommended in clinical suspected cases with an initially negative result. According to the current status, blocking transmission, isolation, respiratory and eye protection, and hand hygiene are the urgent management strategies against 2019-nCoV.

## Data Availability

Not applicable.

## Abbreviations

WHO: World Health Organization
PHEIC: Public health emergency of international concern
SARS: Severe acute respiratory syndrome
MERS: Middle East respiratory syndrome
China CDC: Chinese Center for Disease Control and Prevention
ARDS: Acute respiratory distress syndrome
AI: Artificial Intelligence
ECMO: Extracorporeal membrane oxygenation
TCM: Traditional Chinese medicine
